# Etymologia: Marburg Virus

**DOI:** 10.3201/eid2310.ET2310

**Published:** 2017-10

**Authors:** Ronnie Henry, Frederick A. Murphy

**Keywords:** etymologia, Marburg virus, viruses, viral hemorrhagic fever, outbreak, laboratory workers, African green monkeys, kidneys, Marburg, Germany, Belgrade, Yugoslavia, Angola, Democratic Republic of the Congo

## Marburg [mahrʹboork] Virus

In August and September 1967, an outbreak of a viral hemorrhagic fever occurred among laboratory workers in Marburg and Frankfurt, Germany, and Belgrade, Yugoslavia (now Serbia) who were processing kidneys from African green monkeys that had been imported from Uganda. (These kidneys were used in the production of polio vaccine.) Of 25 primary and 6 secondary cases, 7 were fatal.

A new virus, named Marburg virus, was isolated from patients and monkeys, and the high case-fatality ratio called for the best biocontainment of the day (Figure). The Centers for Disease Control and Prevention (CDC) borrowed a mobile containment laboratory from the National Institutes of Health and set it up in the CDC parking lot; it provided approximately biosafety level 2+ containment. A few isolated, sporadic cases were reported in the following decades until a 1998 outbreak in the Democratic Republic of the Congo affected 154 people with a case-fatality ratio of 83%, and a 2004 outbreak in Angola affected 227 people with a case-fatality ratio of 90%.

**Figure:**
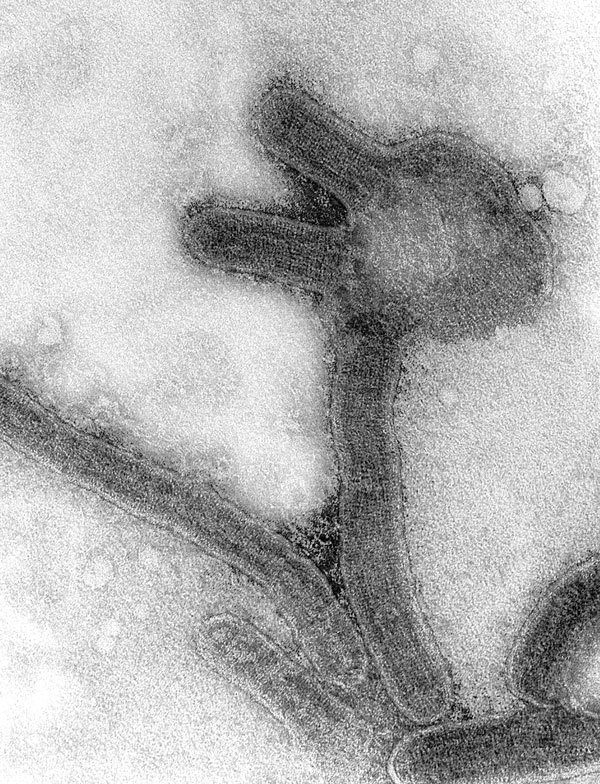
Negative contrast electron microscopy of Marburg virus, from original monkey kidney cell culture propagation done at CDC in 1967, magnification ≈40,000x. Image courtesy of Frederick A. Murphy.
